# ﻿New species of the genus *Pseudocuneopsis* Huang, Dai, Chen & Wu, 2022 (Bivalvia, Unionidae) from Guangxi Province, China

**DOI:** 10.3897/zookeys.1166.104150

**Published:** 2023-06-12

**Authors:** Ruiwen Wu, Lili Liu, Liping Zhang, Junli Jia, Dandong Jin, Xiaoping Wu, Xiongjun Liu

**Affiliations:** 1 School of Life Science, Shanxi Normal University, Taiyuan 030031, China Shanxi Normal University Taiyuan China; 2 Datian High School, Linhai 317004, China Datian High School Linhai China; 3 School of Life Sciences, Nanchang University, Nanchang 330031, China Nanchang University Nanchang China; 4 School of Life Science, Jiaying University, Meizhou 514015, China Jiaying University Meizhou China

**Keywords:** Bivalves, COI, freshwater mussel, morphology, taxonomy

## Abstract

A new species of freshwater mussel belonging to the genus *Pseudocuneopsis*, namely *Pseudocuneopsisyangshuoensis***sp. nov.**, is diagnosed and described from Guangxi Province, China. This paper provides a detailed morphological description, photograph of the type specimen, and anatomical characteristics along with partial sequences of mitochondrial COI as DNA barcode data for this novel species. The new species can be distinguished from its congeners (*Pseudocuneopsissichuanensis* and *Pseudocuneopsiscapitata*) by shell shape, beak position and surface sculpture. The interspecies genetic distance based on the COI barcode between *P.yangshuoensis***sp. nov.** and *P.sichuanensis* is 8%, while it reaches 9% with *P.capitata*. Therefore, we provide robust morphological and molecular evidence to support the validity of this new species.

## ﻿Introduction

Unionidae Gray, 1840 is a family of bivalves (Mollusca: Bivalvia: Unionida) commonly referred to as freshwater mussels (Lopes-Lima et al. 2014; Graf and Cummings 2021). These bivalves are essential members of freshwater ecosystems, playing a variety of ecosystem services, such as nutrient cycling, increasing water purification, bioturbation and habitat provisioning (Vaughn 2018).

China is considered to be one of the major biodiversity hotspots for freshwater mussels, with an abundance of rivers and lakes that harbor a wealth of endemic species (Zieritz et al. 2018; Liu et al. 2022). However, field investigation and research on unionids are concentrated in the middle and lower reaches of the Yangtze River (Wu et al. 2018; Huang et al. 2019; Liu et al. 2020, 2022), with less sampling in the other river basins in Southwest China, for example, Li River in Guangxi Province. These under-investigated areas severely limit the ability to discover new species, which hinders a comprehensive understanding of the phylogeny and evolution within this group.

The genus *Pseudocuneopsis* Huang, Dai, Chen & Wu, 2022 was recently established by Wu et al. (2022a). Based on mitochondrial phylogenomic analyses, Wu et al. (2022a) confirmed that the original genus *Cuneopsissensu lato* Simpson, 1900 was polyphyletic, and proposed two new genera: *Arcuneopsis* and *Pseudocuneopsis*. Later, the genus name *Arcuneopsis* was replaced by *Tchangsinaia* Starobogatov, 1970 because *Uniopiscicula* Heude, 1874 as the type species had previously been classified by Starobogatov (1970) under the genus name *Tchangsinaia*. Currently, the comprehensive molecular systematics have stabilized the taxonomic status of *Pseudocuneopsis*, which is under the subfamily Unioninae in Unionidae (Huang et al. 2019; Wu et al. 2019; Wu et al. 2022a). The genus has two recognized species, i.e., *Pseudocuneopsissichuanensis* Huang, Dai, Chen & Wu, 2022 and *Pseudocuneopsiscapitata* (Heude, 1874); both are endemic to China (Graf and Cummings 2023; MolluscaBase eds 2023). *Pseudocuneopsissichuanensis* has a narrow distribution reported only in the Sichuan Province, and *P.capitata* is widely distributed in the Yangtze River basin (Liu et al. 1979; Liu et al. 2022; Wu et al. 2022a).

In this study, we diagnose and describe a new *Pseudocuneopsis* species from Guangxi Province, China. In addition, we provide estimations of the intraspecific and interspecific genetic distances within *Pseudocuneopsis* based on the mitochondrial COI barcode to examine the species validity.

## ﻿Material and methods

### ﻿Specimen collection, identification and anatomical observations

In December 2022, five samples with tissues were collected from the Li River, Yangshuo County, Guangxi Province, China (24.90099°N, 110.52585°E). All specimens were deposited as vouchers at the Museum of Zoology, Shanxi Normal University (**SXNU**), China (SXNU22121104–SXNU22121108). We performed dissections on all individuals in order to observe soft body characteristics through visual examination and a stereoscopic microscope.

### ﻿DNA extraction and COI amplification

Total genomic DNA was extracted from dissected somatic tissues using TIANamp Marine Animals DNA Kit (Tiangen Biotech, Beijing, China) according to the manufacturer’s instructions.

Polymerase chain reaction (PCR) amplification of the COI gene with a 680-base pair fragment was performed using a primer pair consisting of (LCO22me2 + HCO700dy2) (Walker et al. 2007). Thermal cycling conditions were 98 °C for 10 s, followed by 35 cycles of 94 °C for 1 min, 50 °C for 1 min, 72 °C for 1–2 min, and a final extension of 72 °C for 7 min, following the TaKaRa Ex manufacturer’s protocol. The amplified PCR products were purified and sequenced by Sangon Biotech (Shanghai). The PCR product size for the COI amplicon was 680 bp. The sequences obtained in this study have been uploaded to GenBank (OQ696218–OQ696222).

### ﻿DNA barcode dataset construction

We constructed a mitochondrial COI dataset with the newly obtained sequences from this study and the available *Pseudocuneopsissichuanensis* and *Pseudocuneopsiscapitata* sequences from GenBank. In addition, twenty-five species of the subfamily Unioninae for the ingroups, and two species of the subfamily Gonideinae as the outgroups were downloaded from GenBank and added to the dataset.

As a result, a total of thirty-two COI sequences were used for this study. Sequence details and GenBank accession numbers are shown in Table 1.

**Table 1.** List of sequences used in this study (*) Sequenced from this study.

Taxa	GenBank accession number
UNIONINAE Rafinesque, 1820	
*Lasmigonacompressa* (Lea, 1829)	AF156503
*Pyganodongrandis* (Say, 1829)	AF231734
*Strophitusundulatus* (Say, 1817)	AF156505
*Pseudanodontacomplanata* (Rossmässler, 1835)	KX822661
*Uniotumidus* (Philipsson in Retzius, 1788)	KX822672
*Nodulariadouglasiae* (Griffith & Pidgeon, 1833)	NC_026111
*Aculamprotulascripta* (Heude, 1875)	MF991456
*Aculamprotulatientsinensis* (Crosse & Debeaux, 1863)	NC_029210
*Acuticostachinensis* (Lea, 1868)	MG462919
*Cuneopsisheudei* (Heude, 1874)	MG462974
*Cuneopsisrufescens* (Heude, 1874)	MG462982
*Inversiunioyanagawensis* (Kondo, 1982)	LC518988
*Pseudocuneopsiscapitata* (Heude, 1874)	MZ540968
*Pseudocuneopsiscapitata* (Heude, 1874)	MZ540969
*Pseudocuneopsissichuanensis* Huang, Dai, Chen & Wu, 2022	MZ540966
*Pseudocuneopsissichuanensis* Huang, Dai, Chen & Wu, 2022	MZ540967
*Pseudocuneopsisyangshuoensis* sp. nov. 1*	OQ696218
*Pseudocuneopsisyangshuoensis* sp. nov. 2*	OQ696219
*Pseudocuneopsisyangshuoensis* sp. nov. 3*	OQ696220
*Pseudocuneopsisyangshuoensis* sp. nov. 4*	OQ696221
*Pseudocuneopsisyangshuoensis* sp. nov. 5*	OQ696222
*Tchangsinaiapiscicula* (Heude, 1874)	KJ434496
*Tchangsinaiapiscicula* (Heude, 1874)	KJ434497
*Tchangsinaiapiscicula* (Heude, 1874)	KJ434498
*Tchangsinaiapiscicula* (Heude, 1874)	KJ434499
*Schistodesmuslampreyanus* (Baird & Adams, 1867)	MG463038
*Schistodesmusspinosus* (Simpson, 1900)	MG463045
*Lanceolariagladiola* (Heude, 1877)	KY067441
*Lanceolariagrayii* (Griffith & Pidgeon, 1833)	NC_026686
*Lanceolarialanceolata* (Lea, 1856)	NC_023955
GONIDEINAE Ortmann, 1916	
*Lamprotulaleaii* (Gray in Griffith & Pidgeon, 1833)	NC_023346
*Sinosolenaiaoleivora* (Heude, 1877)	KX822670

COI nucleotide sequences were aligned under the invertebrate mitochondrial code mode in MACSE (Ranwez et al. 2021) with default settings. We calculated and compared inter-and intra-specific distances with MEGA 7.0 (Kumar et al. 2016) using the uncorrected *p*-distance. Standard error was assessed using 1000 bootstrap replicates.

### ﻿Phylogenetic analysis

Bayesian inference (BI) analyses were inferred in MrBayes (Ronquist et al. 2012), using the GTR+I+G model of nucleotide substitution. Four chains were run simultaneously for 10 million generations and trees were sampled every 1000 generations. The first 25% of these trees were discarded as burn-in when computing the consensus tree (50% majority rule). Sufficient mixing of the chains was considered to have been reached when the average standard deviation of split frequencies was below 0.01. Additionally, IQ-TREE was run for maximum likelihood (ML) tree reconstruction with 1000 ultrafast bootstraps (Minh et al. 2013). The ML and BI trees generated are depicted in Suppl. material 1 and Suppl. material 2, respectively.

## ﻿Taxonomy

### ﻿ Pseudocuneopsis yangshuoensis

Taxon classificationAnimaliaUnionidaUnionidae

Wu & Liu sp. nov.

B696928D-8677-5913-B987-EA60ADE2EBAD

https://zoobank.org/8CF6C1AB-184A-40A4-A05B-4B022A3F7EC3

Fig. 1

#### Type specimens.

***Holotype***: China • Guangxi Province, Yangshuo County (阳朔县), Li River (24.90099°N, 110.52585°E), 11 December 2022, coll. Dandong Jin (SXNU22121104). ***Paratypes***: same data as holotype (SXNU22121105 - SXNU22121108).

**Figure 1.**
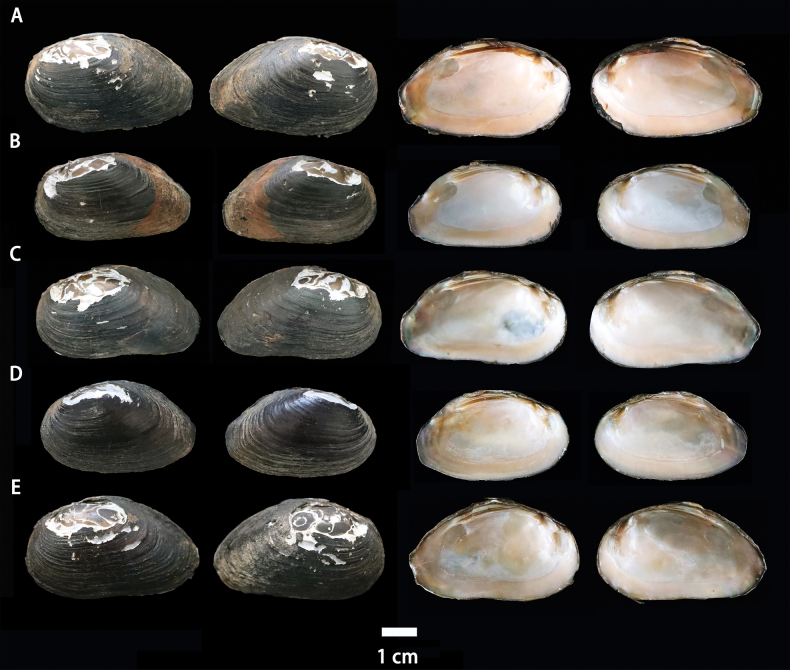
Photographs of *Pseudocuneopsisyangshuoensis* sp. nov. **A** holotype SXNU22121104 **B–E** paratype SXNU22121105–SXNU22121108. Sacle bar: 1 cm.

#### Diagnosis.

*Pseudocuneopsisyangshuoensis* sp. nov. is morphologically distinct from the other two recognized species within the genus by shell shape, beak position and surface sculpture (Table 2). Diagnostic characteristics: shell wedge-shaped; the ventral margin nearly straight or slightly concave; the umbo situated 1/3 of shell length, and obviously lower than the dorsal margin; nacre orange.

**Table 2.** Conchological characters of *Pseudocuneopsisyangshuoensis* sp. nov., *Pseudocuneopsiscapitata*, *Pseudocuneopsissichuanensis*. Characteristic descriptions of *P.capitata* and *P.sichuanensis* are referenced from Wu et al. (2022a).

	*P.yangshuoensis* sp. nov.	*P.sichuanensis*	*P.capitata*
Length	41.39–50.51 (mm)	49.16–62.97 (mm)	101.68–121.32 (mm)
Width	27.25–28.99 (mm)	15.01–22.42 (mm)	37.07–42.72 (mm)
Height	15.34–19.40 (mm)	27.16–36.02 (mm)	49.23–61.02 (mm)
Shell shape	Wedge-shaped	Oval wedge	Elongate wedge
Umbo position	1/3 of shell length; umbo obviously lower than the dorsal margin	1/4–1/5 of shell length; umbo slightly higher than the dorsal margin	1/6 of shell length; umbo obviously higher than the dorsal margin
Surface sculpture	Epidermis brownish-black covered with concentric ridges	Epidermis dark brown with growth annulus with 1 or 2 sulci near the posterior dorsal margin	Epidermis brownish with low rides, which follow the growth lines
Nacre colour	Orange	White	Milk-white
Dorsal margin	Anterior margin oval, and inflated, with the dorsal margin curved downwards	Anterior margin oval, and inflated, with the dorsal margin curved downwards	Anterior margin oval, highly inflated, dorsal margin sloped downwards
Posterior slope	Blunt	Blunt	Sharp
Ventral margin	Nearly straight or slightly concave	Slightly concave inward at middle posterior	Rounded anteriorly, behind the anterior inflation there is a sinus

#### Description.

Shell wedge-shaped, medium thickness; anterior margin regularly rounded and inflated; ventral margin nearly straight or slightly concave; umbo located at 1/3 of shell length and obviously lower than the dorsal margin; umbo sculptured unknown due to severe erosion; posterior slope formed by the ventral margin and dorsal margin low, blunt, approximately 60°; epidermis brownish-black covered with concentric ridges; anterior adductor muscle scars elliptical, deep and unrough; posterior adductor muscle scars long elliptical, shallow and smooth; anterior and posterior retractor muscle scars obvious, with the anterior and posterior irregularly elliptical; mantle muscle scars obvious; left valve with two separate pseudocardinal teeth and two later teeth; the outer and inner pseudocardinal teeth are the same length and projected outward at the same level; right valve with one pseudocardinal tooth and one lateral tooth; lateral and pseudocardinal teeth usually curved; nacre orange in colour.

Length 41.39–50.51 mm, height 15.34–19.40 mm.

#### Etymology.

The specific epithet is derived from the type locality Yangshuo County. For the common name, we recommend “Yangshuo Wedged Mussel” (English) and “Yang Shuo Wei Xie Bang” (阳朔伪楔蚌) (Chinese).

#### Distribution.

**Figure 2.**
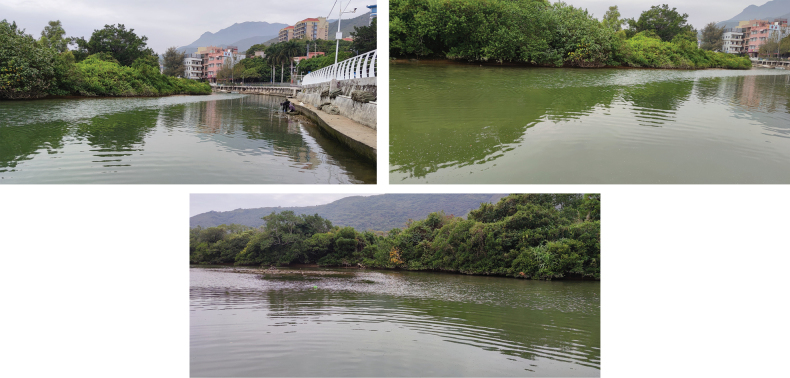
Photograph of the sampling site of *Pseudocuneopsisyangshuoensis* sp. nov. at the Li River, Guangxi in China.

Li River, Guangxi, China (Fig. 2).

#### Anatomical characteristics.

**Figure 3.**
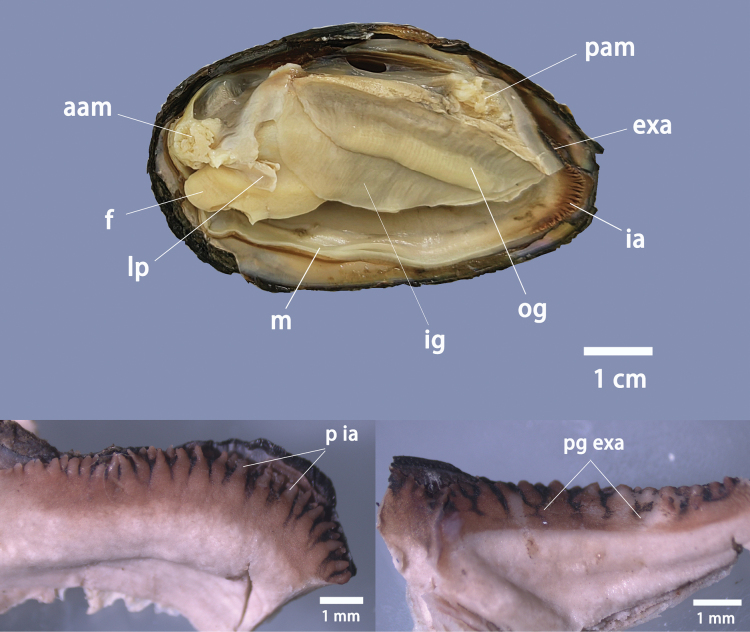
Anatomical features of *Pseudocuneopsisyangshuoensis* sp. nov. with left valve removed. Abbreviations: aam, anterior adductor muscle; pam, posterior adductor muscle; exa, excurrent aperture; ia, incurrent aperture; f, foot; ig, inner gill; og, outer gill; lp, labial palps; m, mantle; p ia, papillae in incurrent aperture; pg exa, pigmentation of excurrent aperture.

The soft tissue morphology reveals elongated papillae arranged in two to three rows within the incurrent aperture, with stocky papillae forming the outer row; notable pigmentation and small sarcomas are present along the outer margin of the excurrent aperture; and the size of inner gills exceeds that of outer gills (Fig. 3).

#### Molecular analyses.

**Figure 4.**
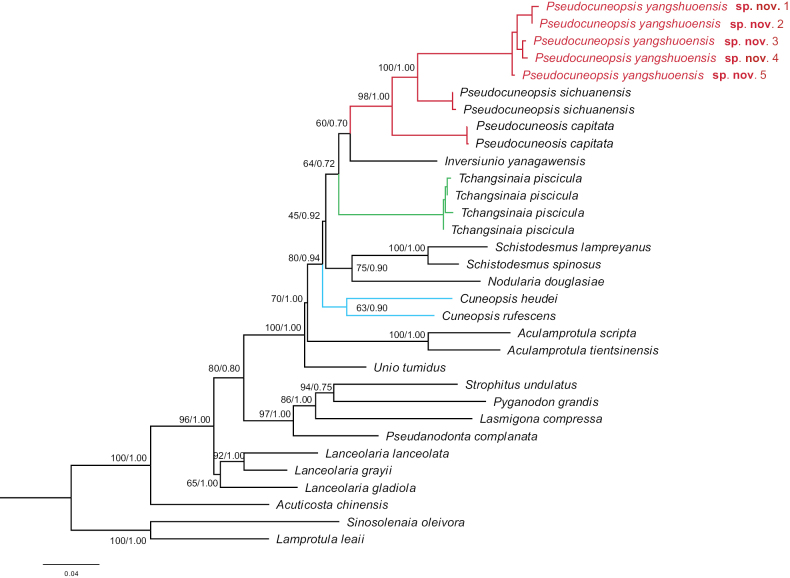
Phylogenetic tree of freshwater mussels inferred from maximum likelihood (ML) and Bayesian inference (BI) analyses of COI barcode. Support values above the branches are bootstrap support (BS)/ posterior probabilities (PP). The red font indicates the species from this study.

Pairwise COI sequence divergences from *Pseudocuneopsisyangshuoensis* sp. nov., *Pseudocuneopsiscapitata*, and *Pseudocuneopsissichuanensis* were conducted in MEGA 7.0 with the uncorrected *p*-distance model. The intraspecific divergence of *Pseudocuneopsisyangshuoensis* sp. nov. was 0%. The interspecific divergence between *Pseudocuneopsisyangshuoensis* sp. nov. and *P.sichuanensis* was 8%, and 9% with *P.capitata*. Both BI and ML trees obtained a consistent topology (Fig. 4). In the phylogenetic trees, *Pseudocuneopsisyangshuoensis* sp. nov. formed a well-supported sister-group relationship with *P.sichuanensis* (BS = 100; PP = 1.00, Fig. 4). *Cuneopsis* and *Tchangsinaia* were each separated from *Pseudocuneopsis* belonging to three different clades (Fig. 4).

#### Remarks.

Species delineation can be problematic in the presence of morphological ambiguities due to phenotypic plasticity and convergence (e.g., cryptic species), especially in mollusks (Zieritz et al. 2010; Inoue et al. 2013). The use of molecular genetics can aid species delineation in the case of phenotypic plasticity and/or convergence (Pieri et al. 2018; Wu et al. 2022b). *Pseudocuneopsisyangshuoensis* sp. nov. can be distinguished from congeneric species based on the diagnostic characteristics of shells. We also analyze the interspecific divergence among *P.sichuanensis*, *P.capitata* and *P.yangshuoensis* sp. nov. based on the COI barcode. The results show that the average interspecific divergence between the two species was 8%–9%, which is much higher than intraspecific divergences. Genetic analysis conducted in this study supports *P.yangshuoensis* sp. nov. as a valid species, which can be easily distinguished by COI barcode.

## Supplementary Material

XML Treatment for Pseudocuneopsis yangshuoensis

## ﻿Additional information

### Author ORCIDs

Ruiwen Wu https://orcid.org/0000-0002-8936-6054

### Data availability

All of the data that support the findings of this study are available in the main text or Supplementary Information.

## Supplementary materials

Supplementary material 1 Phylogenetic tree of freshwater mussels inferred from maximum likelihood (ML) based on COI barcode Data type Phylogenetic tree https://binary.pensoft.net/file/862805This dataset is made available under the Open Database License (http://opendatacommons.org/licenses/odbl/1.0/). The Open Database License (ODbL) is a license agreement intended to allow users to freely share, modify, and use this Dataset while maintaining this same freedom for others, provided that the original source and author(s) are credited.Ruiwen Wu, Lili Liu, Liping Zhang, Junli Jia, Dandong Jin, Xiaoping Wu, Xiongjun Liu

Supplementary material 2 Phylogenetic tree of freshwater mussels inferred from Bayesian inference (BI) analyses based on COI barcode Data type Phylogenetic tree https://binary.pensoft.net/file/862806This dataset is made available under the Open Database License (http://opendatacommons.org/licenses/odbl/1.0/). The Open Database License (ODbL) is a license agreement intended to allow users to freely share, modify, and use this Dataset while maintaining this same freedom for others, provided that the original source and author(s) are credited.Ruiwen Wu, Lili Liu, Liping Zhang, Junli Jia, Dandong Jin, Xiaoping Wu, Xiongjun Liu

## References

[B1] Graf DL Cummings KS (2021) A ‘big data’ approach to global freshwater mussel diversity (Bivalvia: Unionoida), with an updated checklist of genera and species. Journal of Molluscan Studies 87(1): eyaa034. 10.1093/mollus/eyaa034

[B2] Graf DL Cummings KS (2023) The MUSSEL project database. http://www.mussel-project.net/

[B3] Huang XC Su JH Ouyang JX Ouyang S Zhou CH Wu XP (2019) Towards a global phylogeny of freshwater mussels (Bivalvia: Unionida): Species delimitation of Chinese taxa, mitochondrial phylogenomics, and diversification patterns. Molecular Phylogenetics and Evolution 130: 45–59. 10.1016/j.ympev.2018.09.019 30308278

[B4] Inoue K Hayes DM Harris JL Christian AD (2013) Phylogenetic and morphometric analyses reveal ecophenotypic plasticity in freshwater mussels *Obovariajacksoniana* and *Villosaarkansasensis* (Bivalvia: Unionidae). Ecology and Evolution 3(8): 2670–2683. 10.1002/ece3.649 24567831 PMC3930048

[B5] Kumar S Stecher G Tamura K (2016) MEGA7: Molecular Evolutionary Genetics Analysis Version 7.0 for Bigger Datasets. Molecular Biology and Evolution 33(7): 1870–1874. 10.1093/molbev/msw054 27004904 PMC8210823

[B6] Liu YY Zhang WZ Wang QX Wang EY (1979) Economic Fauna of China-Freshwater Mollusk. Science Press, Beijing.

[B7] Liu X Yang X Zanatta DT Lopes-Lima M Bogan AE Zieritz A Ouyang S Wu XP (2020) Conservation status assessment and a new method for establishing conservation priorities for freshwater mussels (Bivalvia: Unionida) in the middle and lower reaches of the Yangtze River drainage. Aquatic Conservation 30(5): 1000–1011. 10.1002/aqc.3298

[B8] Liu XJ Liu YY Wu RW Zanatta DT Lopes-Lima M Gonçalves DV Bogan AE Ouyang S Wu XP (2022) Systematics, distribution, biology, and conservation of freshwater mussels (Bivalvia: Unionida) in China. Aquatic Conservation 32(5): 859–895. 10.1002/aqc.3799

[B9] Lopes-Lima M Teixeira A Froufe E Lopes A Varandas S Sousa R (2014) Biology and conservation of freshwater bivalves: Past, present and future perspectives. Hydrobiologia 735(1): 1–13. 10.1007/s10750-014-1902-9

[B10] Minh BQ Nguyen MAT von Haeseler A (2013) Ultrafast approximation for phylogenetic bootstrap. Molecular Biology and Evolution 30(5): 1188–1195. 10.1093/molbev/mst024 23418397 PMC3670741

[B11] MolluscaBase eds (2023) MolluscaBase. *Pseudocuneopsis* X.-C. Huang, Y.-T. Dai & X.-P. Wu, 2022. https://www.molluscabase.org/aphia.php?p=taxdetails&id=1559799 [Accessed on 2023-05-13]

[B12] Pieri AM Inoue K Johnson NA Smith CH Harris JL Robertson C Randklev CR (2018) Molecular and morphometric analyses reveal cryptic diversity within freshwater mussels (Bivalvia: Unionidae) of the western Gulf coastal drainages of the USA. Biological Journal of the Linnean Society, Linnean Society of London 124(2): 261–277. 10.1093/biolinnean/bly046

[B13] Ranwez V Chantret N Delsuc F (2021) Aligning Protein-Coding Nucleotide Sequences with MACSE. In: Katoh K (Ed.) Multiple Sequence Alignment. Methods in Molecular Biology, vol 2231. Humana, New York, NY. 10.1007/978-1-0716-1036-7_4 33289886

[B14] Ronquist F Teslenko M Mark PV Ayres DL Darling A Höhna S Larget B Liu L Marc A Suchard MA Huelsenbeck JP (2012) MrBayes 3.2: Efficient Bayesian phylogenetic inference and model choice across a large model space. Systematic Biology 61(3): 539–542. 10.1093/sysbio/sys029 22357727 PMC3329765

[B15] Starobogatov Y (1970) Fauna of molluscs and zoogeographic division of continental waterbodies of the globe [In Russian]. Nauka, Leningrad.

[B16] Vaughn CC (2018) Ecosystem services provided by freshwater mussels. Hydrobiologia 810(1): 15–27. 10.1007/s10750-017-3139-x

[B17] Walker JM Bogan AE Bonfiglio EA Campbell DC Christian AD Curole JP Harris JL Wojtecki RJ Hoeh WR (2007) Primers for amplifying the hypervariable, male-transmitted COII–COI junction region in amblemine freshwater mussels (Bivalvia: Unionoidea: Ambleminae). Molecular Ecology Notes 7(3): 489–491. 10.1111/j.1471-8286.2006.01630.x

[B18] Wu RW Liu YT Wang S Liu XJ Zanatta DT Roe KJ Song XL An CT Wu XP (2018) Testing the utility of DNA barcodes and a preliminary phylogenetic framework for Chinese freshwater mussels (Bivalvia: Unionidae) from the middle and lower Yangtze River. PLoS ONE 13(8): e0200956. 10.1371/journal.pone.0200956 PMC6082535 30089124

[B19] Wu RW Liu XJ Wang S Roe KJ Ouyang S Wu XP (2019) Analysis of mitochondrial genomes resolves the phylogenetic position of Chinese freshwater mussels (Bivalvia, Unionidae). ZooKeys 812: 23–46. 10.3897/zookeys.812.29908 PMC6328525 30636909

[B20] Wu XP Dai YT Yin N Shu FY Chen ZG Guo L Zhou CH Ouyang S Huang XC (2022a) Mitogenomic phylogeny resolves *Cuneopsis* (Bivalvia: Unionidae) as polyphyletic: The description of two new genera and a new species. Zoologica Scripta 51(2): 173–184. 10.1111/zsc.12527

[B21] Wu R Liu X Guo L Zhou C Ouyang S Wu X (2022b) DNA barcoding, multilocus phylogeny, and morphometry reveal phenotypic plasticity in the Chinese freshwater mussel *Lamprotulacaveata* (Bivalvia: Unionidae). Ecology and Evolution 12(7): e9035. 10.1002/ece3.9035 PMC9277607 35845369

[B22] Zieritz A Hoffman JI Amos W Aldridge DC (2010) Phenotypic plasticity and genetic isolation-by-distance in the freshwater mussel *Uniopictorum* (Mollusca: Unionoida). Evolutionary Ecology 24(4): 923–938. 10.1007/s10682-009-9350-0

[B23] Zieritz A Bogan AE Froufe E Klishko O Kondo T Kovitvadhi U Kovitvadhi S Lee JH Lopes-Lima M Pfeiffer JM Sousa R Van Do T Vikhrev I Zanatta DT (2018) Diversity, biogeography and conservation of freshwater mussels (Bivalvia: Unionida) in East and Southeast Asia. Hydrobiologia 810(1): 29–44. 10.1007/s10750-017-3104-8

