# Supplementary figures and images for: ﻿New species of the genus Pseudocuneopsis Huang, Dai, Chen & Wu, 2022 (Bivalvia, Unionidae) from Guangxi Province, China
Source: Zookeys. 2023 Jun 12;1166:261–70. doi: 10.3897/zookeys.1166.104150 (PMC10280198; doi:10.3897/zookeys.1166.104150)

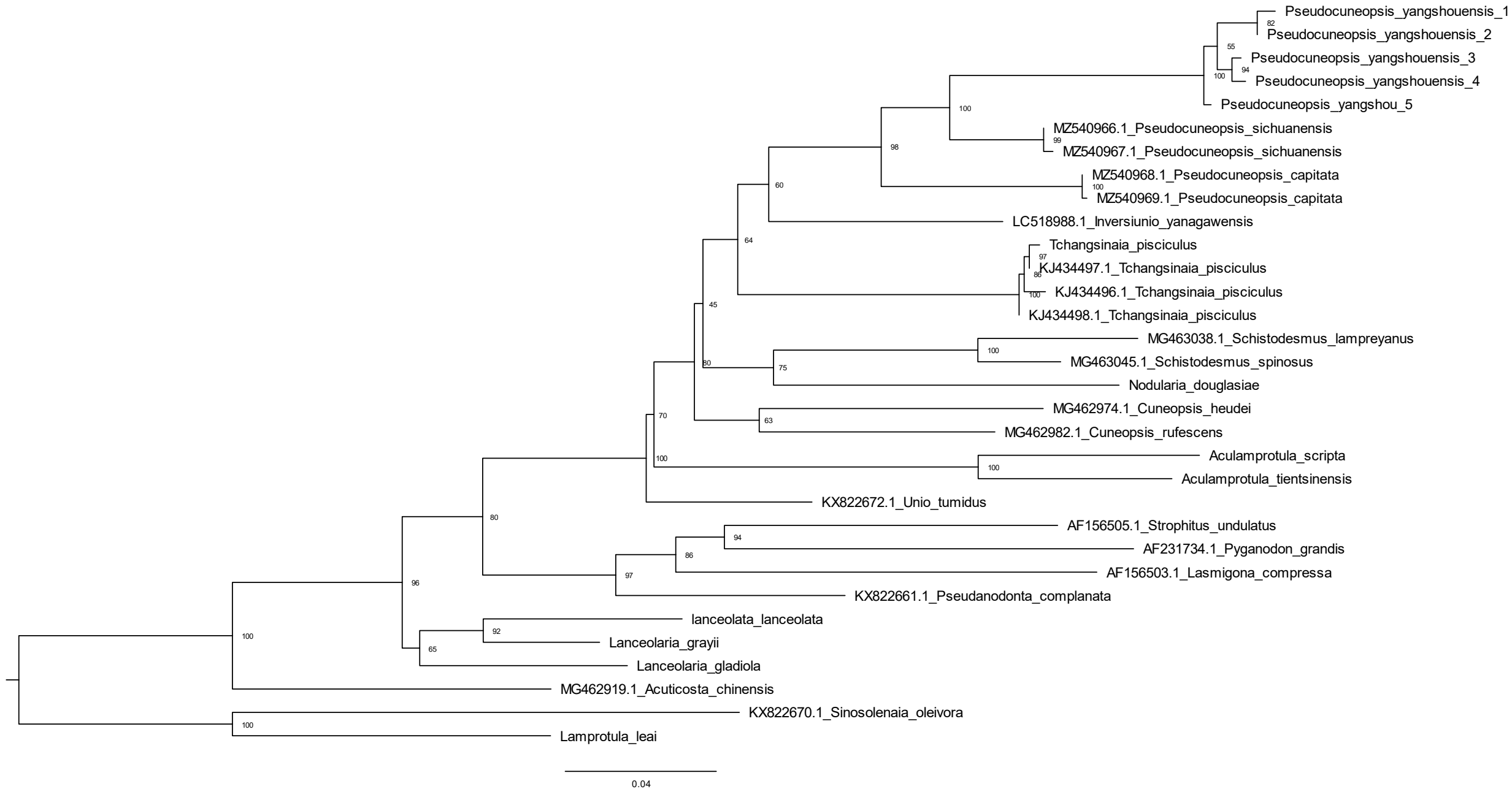

Supplement: Supplementary material 1 — Phylogenetic tree of freshwater mussels inferred from maximum likelihood (ML) based on COI barcode [file zookeys-1166-261_article-104150__-s001.pdf]

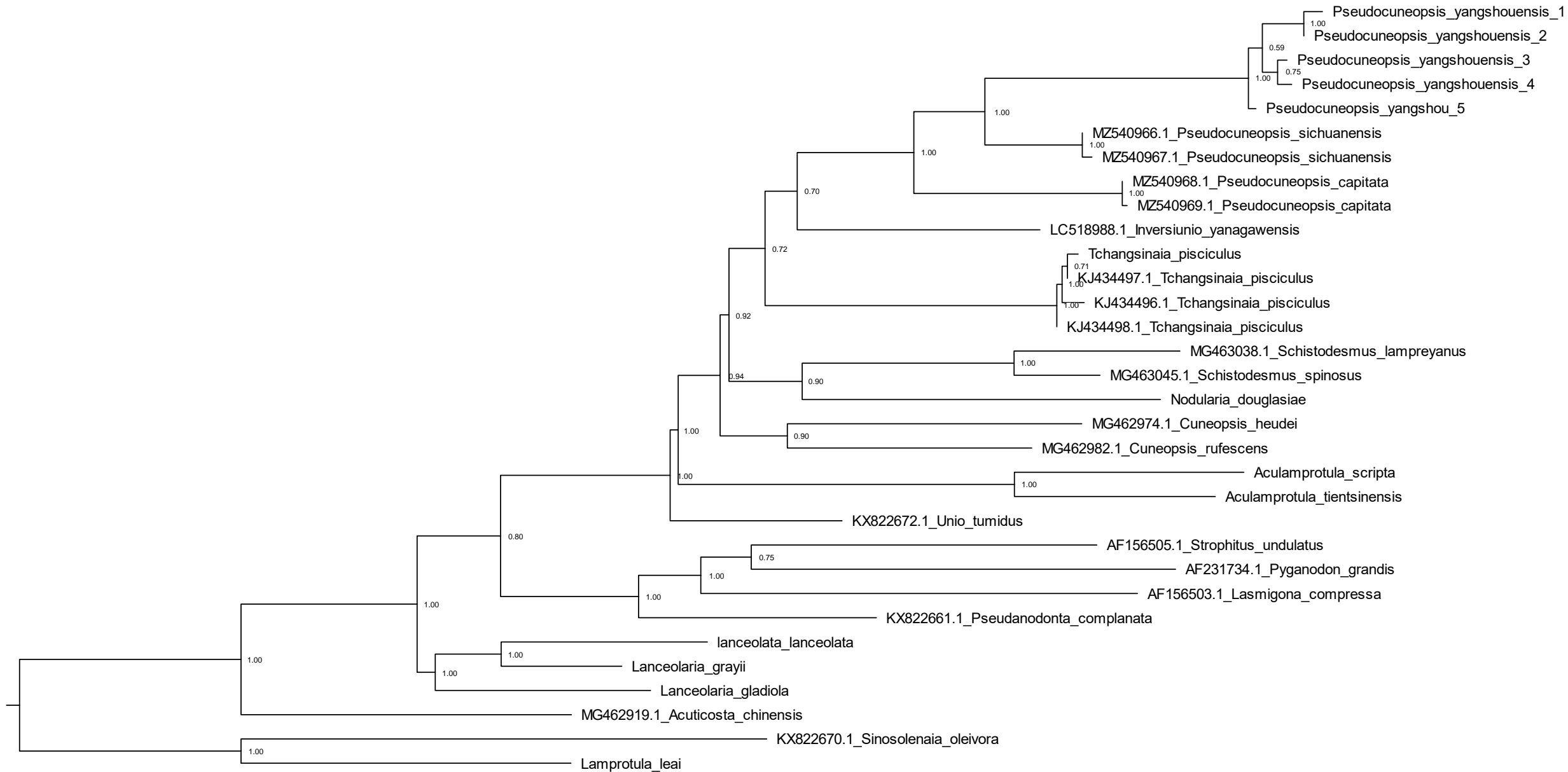

0.2

Supplement: Supplementary material 2 — Phylogenetic tree of freshwater mussels inferred from Bayesian inference (BI) analyses based on COI barcode [file zookeys-1166-261_article-104150__-s002.pdf]
